# Megacystis-Microcolon-Intestinal Hypoperistalsis Syndrome: A Case Report of an Uncommon Condition

**DOI:** 10.7759/cureus.54255

**Published:** 2024-02-15

**Authors:** Marcia Mejia, Mónica Royero Arias, Jonathan Pimiento Figueroa, Walter Romero Espitia

**Affiliations:** 1 Radiology, Universidad de Antioquia, Medellín, COL; 2 Pediatric Radiology, Hospital Universitario San Vicente Fundación, Medellín, COL; 3 Radiology, Hospital Universitario San Vicente Fundación, Medellín, COL; 4 Pediatric Surgery, Hospital Universitario San Vicente Fundación, Medellín, COL

**Keywords:** microcolon, pediatric surgery, diagnostic imaging, intestinal pseudo-obstruction, megacystis microcolon intestinal hypoperistalsis syndrome

## Abstract

The megacystis-microcolon-intestinal hypoperistalsis syndrome (MMIHS), also known as Berdon syndrome, is a rare congenital condition that falls within the spectrum of visceral myopathies. It is characterized by the presence of megacystis, microcolon, and hypoperistalsis, which are secondary to gastrointestinal and urinary system dysmotility. It is frequently associated with other alterations in the gastrointestinal and genitourinary tracts. Although it is possible to make the diagnosis in the prenatal period, most cases are diagnosed after birth through genetic and imaging studies. Advances in treatment have led to a progressive increase in survival rates. We present the case of a newborn with congenital alterations described prenatally and with imaging findings characteristic of the syndrome.

## Introduction

The megacystis-microcolon-intestinal hypoperistalsis syndrome (MMIHS) or Berdon syndrome is a rare condition, first described in 1976, with 450 reported cases in the literature up to 2019 [1,2]. It is part of the spectrum of intestinal myopathies, with Hirschsprung's disease being the most common [3]. It is characterized by gastrointestinal and genitourinary dysmotility, resulting in its main manifestations: megacystis, microcolon, and decreased peristalsis. Additionally, it is often accompanied by multiple alterations, especially gastrointestinal and genitourinary, such as esophageal dilatation, stomach dilatation, dilated loops of the small intestine, intestinal malrotation, urinary tract dilatation, and vesicoureteral reflux [4-9]. Most patients are diagnosed after birth, but prenatal diagnosis is possible in up to 25% of cases based on ultrasound findings [4,8]. Imaging studies are essential for diagnosis, but genetic testing is necessary to detect underlying mutations that may influence prognosis and family counseling [2].

The prognosis of this condition has evolved over time. In the early years following its description, the mortality rate within the first year of life was over 90%. However, with advances in treatments and the implementation of intestinal or multivisceral transplantation in selected patients, there has been an increase in survival rates [7,10,11].

## Case presentation

We present the case of a female premature newborn with congenital abnormalities of the urinary tract detected *in-utero*. A prenatal ultrasound at 23 weeks showed an increase in the transverse diameter of the bilateral renal pelvis (8 mm on the right and 9 mm on the left), megacystis (15 mm craniocaudal diameter), and dysplastic renal parenchyma. An *in-utero* vesicoamniotic shunt procedure was performed, but the results of the amniotic fluid analysis are not available. She was born at 32 weeks by cesarean section due to the detected malformations, weighing 2470 grams (97th percentile). On the first day of life, she showed symptoms of intestinal obstruction such as abdominal distension, oral intolerance, and inability to pass meconium. An exploratory laparotomy on day 2 of life revealed intestinal malrotation, volvulus, and microcolon. Ladd's procedure was performed for the malrotation, along with gastrostomy and cystostomy for decompression. Parenteral nutrition was initiated without oral or gastrostomy feeding.

She developed persistent bacteremia and malnutrition, leading to a referral to a higher-level hospital in the fourth week of life. On admission, she exhibited low weight and height (2nd and 9th percentile, respectively) and generalized jaundice on physical examination. Laboratory tests revealed cholestasis and anemia: Alkaline phosphatase at 244 IU/L, Aspartate transaminase at 41 IU/L, Alanine aminotransferase at 16 IU/L, total bilirubin at 9.76 mg/dl, direct bilirubin at 7.6 mg/dl, and hemoglobin at 9.7 g/dl.

**Figure 1:**
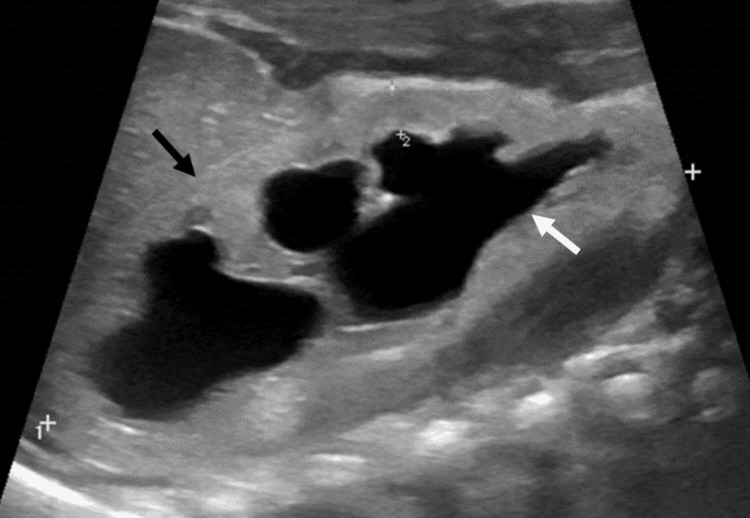
Ultrasound showing dilation of central and peripheral calyces (white arrow) with thinning and dysplastic changes of the renal parenchyma (black arrow).

She received multidisciplinary care from different specialties (pediatric surgery, pediatric nephrology, pediatric neurology, pediatric radiology, genetics, and nutrition), and imaging studies were requested. Abdominal ultrasound showed pelvicalyceal and ureteral dilatation and dysplastic changes in the bilateral renal parenchyma (Figure 1).

**Figure 2:**
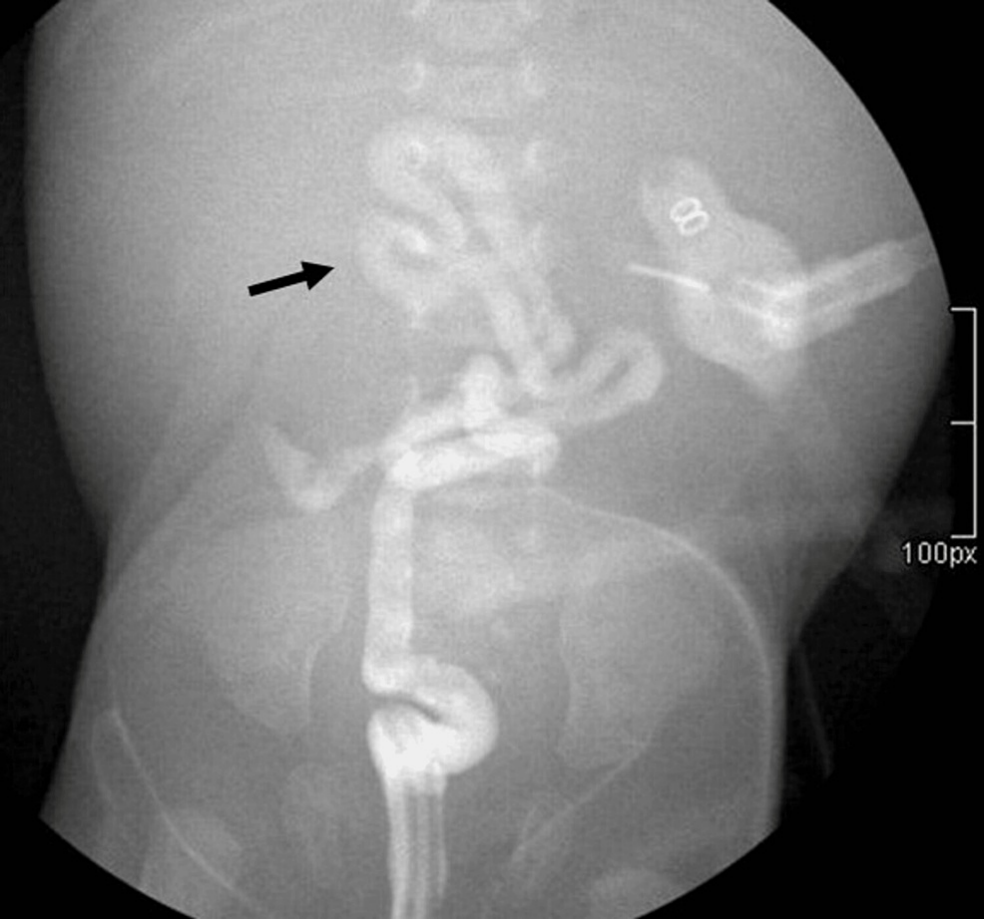
Barium enema showing centralized colon with decreased caliber and loss of haustral pattern due to microcolon (black arrow).

A barium enema revealed decreased colonic caliber with loss of haustral pattern, indicative of microcolon (Figure 2).

**Figure 3:**
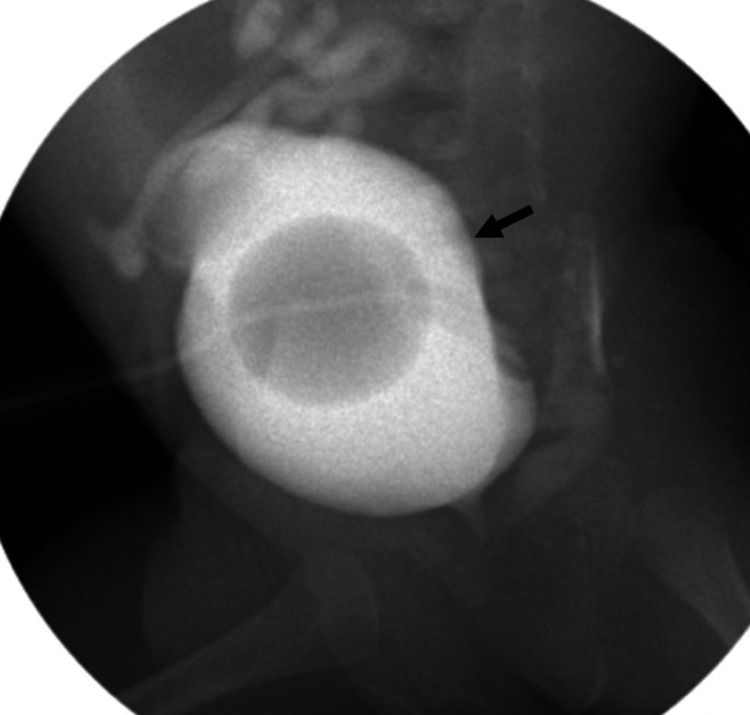
Cystography through cystostomy tube filling bladder with more than double the vesical capacity (black arrow). No vesicoureteral reflux was observed. Residual contrast medium in colon.

Cystography through the cystostomy tube demonstrated bladder dilatation with filiform contrast passage through the urethra after 20 minutes of bladder filling (Figure 3).

**Figure 4:**
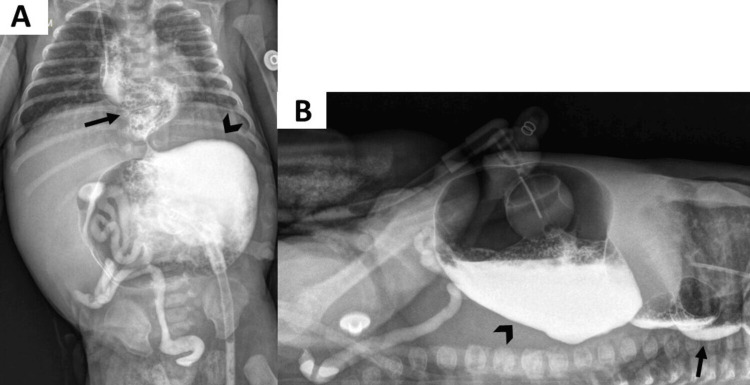
Upper gastrointestinal series through gastrostomy tube, AP projection (A) and horizontal beam (B) demonstrating marked dilation of the stomach (arrowhead) without contrast medium passage to the duodenum, with supracarinal gastroesophageal reflux revealing tortuous and dilated esophagus (black arrow). Residual contrast medium was noted in the colon.

Upper gastrointestinal series showed megaesophagus, gastroesophageal reflux, and stomach dilatation without contrast passage to the duodenum (Figure 4).

Based on the aforementioned findings, Berdon syndrome was suspected, with cholestasis secondary to parenteral nutrition. It was deemed that the patient was not a candidate for further surgical procedures with the available resources. She was discharged to her city of origin at 7 weeks of age with supportive management and total parenteral nutrition. She was never fed orally or through gastrostomy, and also did not have spontaneous pass of stools.

## Discussion

Megacystis-microcolon-intestinal hypoperistalsis syndrome is a rare congenital condition that occurs more frequently in women (with a female-to-male ratio of 2-4:1) but presents with greater severity in men [4,7]. Its etiopathogenesis has not been fully understood. However, an alteration in the smooth muscle architecture is proposed as one of its main causes, without specific findings in the histopathological study [3,12].

Different autosomal dominant and recessive genetic mutations associated with this syndrome have been described; however, in up to 50% of patients, the underlying alteration cannot be identified [2,13]. The mutation in the ACTG2 gene is the most frequent (88% of cases); it shows autosomal dominant inheritance and it generates the most severe phenotype. Autosomal recessive mutations involve the genes MYH11, LMOD1, and MYL9. Cases of the syndrome with a familial association have been reported; however, the majority of cases occur sporadically [2].

Diagnosis of MMIHS can be suspected from the prenatal period due to the presence of megacystis on ultrasound, which is found in up to 88% of patients [5,14]. The craniocaudal diameter of the bladder in a sagittal section is normal up to 6 mm in weeks 10-14 of gestation; megacystis is considered when it is greater than 7 mm or when it exceeds 10% of the total craniocaudal diameter of the fetus. There is no strict definition of megacystis in the second and third trimesters of gestation. However, it has been proposed that the absence of bladder emptying after 45 minutes can be a marker of megacystis during gestation [15]. Other findings described in prenatal imaging are dilations of the urinary tract and stomach, with normal amniotic fluid or with polyhydramnios [8,14]. In vesicocentesis, the concentrations of sodium and chloride will be normal, suggesting preserved renal function [10].

After birth, patients will present clinical manifestations secondary to functional obstruction of the gastrointestinal and genitourinary tracts, which include abdominal distension, bilious vomiting, failure to pass meconium, inability for spontaneous voiding requiring catheterization, and decreased or absent peristalsis [5].

The main findings of the syndrome are usually accompanied by a spectrum of additional abnormalities that can affect multiple systems. The most frequent are intestinal malrotation, vesicoureteral reflux, and omphalocele [4,9]. Megaesophagus is rarely mentioned within the clinical findings of the syndrome; however, some authors consider that this may be the result of disease involvement or secondary to functional obstruction of the gastrointestinal tract [16,17].

Imaging studies will be fundamental for the definitive diagnosis. The X-ray will show stomach dilation with or without intestinal loop dilation, and the absence of distal gas. Cystography may reveal megabladder and vesicoureteral reflux. Fluoroscopic studies of the upper digestive tract may show stomach dilation, delayed or absent gastric emptying and transit to the small bowel, intestinal loops dilation, and intestinal malrotation. Megaesophagus will manifest as tortuosity and subjective increase in caliber, often accompanied by gastric reflux. In the barium enema, there will be microcolon, which is manifested by decreased caliber with a usually normal length and alteration in the pattern of haustras [4,5,12]. There is no definitive measure to define microcolon; however, some authors have proposed that a lumen diameter smaller than the height of a lumbar vertebra can be considered microcolon [18].

The main differential diagnoses are Prune Belly Syndrome, Hirschsprung's disease, posterior urethral valves, and chronic intestinal pseudo-obstruction (CIPO). In the first, there are additional findings such as alterations of the abdominal wall, cryptorchidism, and oligohydramnios. In Hirschsprung's disease, the absence of ganglion cells in the rectal biopsy will be the key to diagnosis. In the case of posterior urethral valves, there is no gastrointestinal involvement, and in CIPO microcolon is not found [3,5,9].

The management of this syndrome requires a multidisciplinary approach. Patients initially require parenteral nutrition, gastrointestinal decompression through gastrostomies or enterostomies, as well as bladder decompression through vesicostomy or bladder catheterization. In addition to intestinal rehabilitation, intestinal or multivisceral transplantation is becoming increasingly common in experienced centers [7,9]. The multivisceral transplant may include the stomach, pancreatoduodenal complex, spleen, kidneys, and intestines [19]. The prognosis for these patients has evolved, with survival rates increasing from less than 10% in the first year of life to over 90% in a series of cases with transplanted patients followed for 10 years [7]. The main causes of death are malnutrition, sepsis, renal failure, and liver failure secondary to parenteral nutrition [9-11].

## Conclusions

MMIHS is a rare condition. Some findings on prenatal ultrasound may raise suspicion of the diagnosis; however, postnatal imaging studies in conjunction with genetic testing are necessary in most cases to confirm it. Therapeutic advances have improved survival, especially through intestinal or multivisceral transplantation in selected patients. Early detection can allow for appropriate counseling of parents and timely treatment planning. Our case is an example of the characteristic clinical and imaging findings of the syndrome in a female newborn.
